# Molecular barcode and morphological analysis of *Smilax purhampuy* Ruiz, Ecuador

**DOI:** 10.7717/peerj.11028

**Published:** 2021-03-18

**Authors:** Pilar Soledispa, Efrén Santos-Ordóñez, Migdalia Miranda, Ricardo Pacheco, Yamilet Irene Gutiérrez Gaiten, Ramón Scull

**Affiliations:** 1 Facultad de Ciencias Químicas. Ciudadela Universitaria “Salvador Allende”, Universidad de Guayaquil, Guayaquil, Ecuador; 2 Facultad de Ciencias de la Vida, Campus Gustavo Galindo, ESPOL Polytechnic University, Escuela Superior Politécnica del Litoral, ESPOL, Guayaquil, Ecuador; 3 Centro de Investigaciones Biotecnológicas del Ecuador, Campus Gustavo Galindo, ESPOL Polytechnic University, Escuela Superior Politécnica del Litoral, ESPOL, Guayaquil, Ecuador; 4 Facultad de Ciencias Naturales y Matemáticas, ESPOL Polytechnic University, Escuela Superior Politécnica del Litoral, ESPOL, Guayaquil, Ecuador; 5 Instituto de Farmacia y Alimentos, Universidad de La Habana, Ciudad Habana, Cuba

**Keywords:** *atpF-atpH* spacer, *matK*, *rbcL*, *rpoB*, *rpoC1*, *psbK*–*psbI* spacer, ITS2, Medicinal

## Abstract

Smilax plants are distributed in tropical, subtropical, and temperate regions in both hemispheres of the world. They are used extensively in traditional medicines in a number of countries. However, morphological and molecular barcodes analysis, which may assist in the taxonomic identification of species, are lacking in Ecuador. In order to evaluate the micromorphological characteristics of these plants, cross sections of *Smilax purhampuy* leaves were obtained manually. The rhizome powder, which is typically used in traditional medicines, was analyzed for micromorphological characteristics. All samples were clarified with 1% sodium hypochlorite. Tissues were colored with 1% safranin in water and were fixed with glycerinated gelatin. DNA was extracted from the leaves using a modified CTAB method for molecular barcode characterization and PCR was performed using primers to amplify the different *loci* including the plastid genome regions *atpF-atpH* spacer, *matK* gene, *rbcL* gene, *rpoB* gene, *rpoC1* gene, *psbK–psbI* spacer, and *trnH–psbA* spacer; and the nuclear DNA sequence ITS2. A DNA sequence similarity search was performed using BLAST in the GenBank nr database and phylogenetic analysis was performed using the maximum likelihood method according to the best model identified by MEGAX using a bootstrap test with 1,000 replicates. Results showed that the micromorphological evaluation of a leaf cross section depicted a concave arrangement of the central vein, which was more pronounced in the lower section and had a slight protuberance. The micromorphological analysis of the rhizome powder allowed the visualization of a group of cells with variable sizes in the parenchyma and revealed thickened xylematic vessels associated with other elements of the vascular system. Specific amplicons were detected in DNA barcoding for all the barcodes tested except for the *trnH–psbA* spacer. BLAST analysis revealed that the *Smilax* species was predominant in all the samples for each barcode; therefore, the genus *Smilax* was confirmed through DNA barcode analysis. The barcode sequences *psbK-psbI, atpF-atpH,* and ITS2 had a better resolution at the species level in phylogenetic analysis than the other barcodes we tested.

## Introduction

The genus *Smilax* (in the family Smilacaceae) consists of 310 species that are distributed in tropical, subtropical and temperate regions in both hemispheres of the world (Qi et al., 2013). According to Cameron & Fu (2006), Smilacaceae are taxonomically confused and belong to the cosmopolitan family of Liliales. Due to morphological analysis, the division of Smilacaceae includes at least seven genera and five sections within the large genus *Smilax*. Plants are dioecious, vine, herbaceous, or rarely, sub-shrubs or shrubs. Leaves are simple, and alternating with petioles that have tendrils; the primary venation are acrodomous. Thicker stems are rippled while aerial stems are generally aculeate (Martins et al., 2013b). In Ecuador, the genus is not well-recorded, although approximately ten species of this genus have been reported in the country according to Gaskin (1999).

Smilax is used a variety of ways in traditional medicine. For instance, in Brazil, *Smilax longifolia* Rich and *Smilax syphilitica* Humb & Bonpl. ex Wild are used as diuretics and in the treatment of venereal diseases (Breitbach et al., 2013) and *Smilax quinquenervia* Vell is used as a tonic for rheumatism and as an anti-syphilitic (Andreata, 1997). In Central America, several species of *Smilax* are used as diuretics, and for dermatological infections, gastrointestinal disorders, rheumatism, vaginitis, contraception, menstrual regulation, anemia, snake bites, and arthritis. In Ecuador, *Smilax* species are used for the elimination of cholesterol and triglycerides, the treatment of arthritis, intestinal, stomach and prostate inflammations, chronic gastritis, and cysts (Ferrufino & Gómez, 2004). Several pharmacological properties have been demonstrated, including glucose-lowering (Romo-Pérez, Escandón-Rivera & Andrade-Cetto, 2019), anti-hyperuricemic (Huang et al., 2019), anti-inflammatory and analgesic (Khana et al., 2019), diuretic (Pérez-Ramírez et al., 2016), and antioxidant (Fonseca et al., 2017) effects. Several chemical compounds have been identified in the genus, including polysaccharides (Zhang, Pan & Ran, 2019), steroidal saponins (Luo et al., 2018), and flavonoids (Wang et al., 2019), among others.

*Smilax purhampuy* is native to the Amazon and is distributed throughout Ecuador, Peru, Nicaragua, Colombia, Bolivia, Costa Rica, Venezuela, Honduras, and Brazil (Rivas et al., 2017). *S. purhampuy* is traditionally known for its healing and therapeutic properties. It has been used to treat cholesterol and triglycerides, chronic gastritis, cysts, arthritis, and intestinal, stomach, and prostate inflammations (JR Global del Perú, 2011). However, information on *S. purhampuy* is limited.

Morphological and molecular barcode analysis are lacking for *S. purhampuy* in Ecuador, despite its medicinal use. Phylogenetic analysis of the *Smilax* genus includes microsatellites (Martins et al., 2013a; Ru et al., 2017; Qi et al., 2017) which are also informative tools for genetic diversity and gene flow studies. Other methods for the phylogenetic analysis of plants includes DNA barcodes, which may be used as a complementary tool in the taxonomic identification of the species; for instance, the plastid genome regions *atpF-atpH* spacer, *matK* gene, *rbcL* gene, *rpoB* gene, *rpoC1* gene, *psbK–psbI* spacer, and *trnH–psbA*  spacer have been tested as universal plant barcodes (CBOL Plant Working Group, 2009). The chloroplast genes *rbcL* and *matK*, are recommended to characterize land plants as a 2-*locus* combination (CBOL Plant Working Group, 2009).

The first reported use of the DNA barcode in *Smilax* species included the rDNA ITS sequence (Cameron & Fu, 2006) inferring phylogenetic relationships that elucidated the evolutionary and biogeographic history of the genera from the Smilacaceae family.

Sulistyaningsih et al. (2018) used the DNA barcode *rbcL* for phylogenetic analysis of *Smilax* spp. in Java, Indonesia. Qi et al. (2013) used the DNA barcodes ITS, *matK* and the *rpl16* intron in Smilacaceae indicating that the phylogenetic relationships largely contradicted the traditional morphological classification of the family. Wang et al. (2014) used the DNA barcode *psbA-trnH* to distinguish *Smilax glabra* from its related species, and Kritpetcharat et al. (2011) used the *trnH-psbA* spacer barcode for *Smilax china* and *S. glabra* indicating that measuring the genetic distance may be used to discriminate between the two species. In a broader study which includes four species of *Smilax* and other trees, Liu et al. (2015) used the DNA barcodes *rbcL*, *matK*, ITS, ITS2, and *trnH-psbA* to analyze the diversity and species resolution, concluding that the combination of the *loci rbcl* + ITS2 is an effective tool for documenting plant diversity in the Dinghushan National Nature Reserve in China. Other *loci* in medicinal plants have been proposed for their characterization, including the nuclear sequence ITS2 (Zhang et al., 2016). Furthermore, DNA barcodes could be used to distinguish adulterated drugs (Kumari & Kotecha, 2016). Therefore, DNA barcode analysis should be performed for *Smilax* species to verify the results of taxonomic and morphological studies. We investigated the micromorphological and molecular barcode characterization of *S. purhampuy* Ruiz collected in Ecuador and found that the barcodes *psbK-psbI, atpF-atpH,* and ITS2 could be used in *Smilax* plants for a better resolution at the species level.

## Materials and Methods

### Study area

The climate of the study area is rainy megathermal with an average monthly temperature between 22 °C and 26 °C and average rainfall between 2,000 to 3,000 mm per year. The study area is a tropical humid forest.

### Collection of plant material

Plant material was collected from three specimens of *S. purhampuy* Ruiz in the Francisco de Orellana Province in Ecuador (coordinates 1°10′03.7″S 76°56′30.9″W) in March and April of 2019. The samples were collected from shaded-exposed plants. Branches containing leaves, fruits, and the rhizome were transferred to the GUAY herbarium of the Faculty of Natural Sciences of the University of Guayaquil for taxonomic characterization. Samples were identified as *S. purhampuy* Ruiz (voucher number 13,117).

### Plant material preparation

Leaves and rhizomes were washed with water. The leaves used in the micromorphological study were analyzed and stored at −80 °C for DNA extraction. The rhizomes were dried in a Mettler Toledo stove at 40 °C and then samples of the rhizomes were crushed with a manual knife mill and stored in amber glass jars for analysis.

### Micromorphological analysis

Leaf samples were taken from the middle of the lamina for the evaluation of their micromorphological characteristics. The mid-rib was cut transversely according to the manual method (Miranda & Cuéllar, 2000). The maximum sample width was 1 cm including the mid-rib. Transversal cuts of fresh leaves were hydrated and clarified with 1% sodium hypochlorite. Tissues were colored with 1% safranin in water and fixed with glycerinated gelatin according to the method by Gattuso & Gattuso (1999). The powder obtained from the rhizomes was hydrated, clarified with 1% sodium hypochlorite and colored with 1% safranin in water and fixed with glycerinated gelatin (Gattuso & Gattuso, 1999; Miranda & Cuéllar, 2000). We performed a histochemical reaction with Lugol reagent to detect starch in the powdered drug obtained from the rhizomes (Gattuso & Gattuso, 1999). Morphological analysis was performed using a NOVEL light microscope at 10x magnification, attached to an HDCE-50B digital camera, model 146 HDCE-50B.

### DNA extraction and PCR

Leaf samples from the three *S. purhampuy* Ruiz plants (with codes CIBE-010, CIBE-011, CIBE-012) were ground with MM400 (Retsch, Haan, Germany) and liquid nitrogen and were stored at −80 °C. DNA extraction was performed for each *Smilax* plant independently. A modified CTAB protocol was used for total DNA extraction according to Pacheco Coello et al. (2017). The master mix GoTaq® 2x (Cat# M7123, Promega) was used for PCR analysis according to the manufacturer’s instructions using 0.5 µM for each primer according to the barcode used (Table S1) in a 50 µL PCR reaction. The conditions for the PCR were: 95 °C for 3 min for initial denaturation; 35 cycles of 95 °C for 30 s, 50 °C/56 °C/60 °C (depending of the barcode, Table S1) for 60 s, 72 °C for 60 s; and a final extension of 72 °C for 10 min. Amplicons were detected by sampling 5 µL in agarose gel (1.5%) electrophoresis. The remaining 45 µL was purified and sequenced commercially (Macrogen, Rockville, MD, USA). At least two technical replicates were sequenced and a consensus was generated for each biological replicate.

### Bioinformatic analysis

Sequences were processed using MEGAX (Stecher, Tamura & Kumar, 2020). Technical replicates were aligned with MUSCLE and a consensus sequence was generated for each barcode. Consensus sequences were analyzed by BLAST (Zhang et al., 2000) in the GenBank non-redundant nucleotide database (nr). The nr database included the accessions of the complete plastid genomes of *Smilax* spp. representing three species and also accessions containing sequences of single locus, indicating that the results were dependent on the sequence availability in the database at the time of the analysis (28th June 2020). Accessions were selected for phylogenetic analysis based on BLAST analysis. For each barcode, the accessions and samples sequences were aligned using MUSCLE and the recommended model from MEGAX was used. The aligned sequences were trimmed at the ends to allow for all sequences to maintain the same range. The maximum likelihood method was performed according to the best model found by MEGAX using bootstrap test (1,000 replicates).

## Results

### Morphological analysis

**Figure 1:**
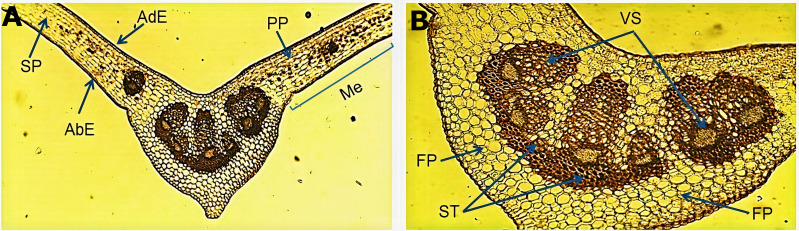
Microscopic characteristics of the leaf from *Smilax purhampuy* Ruiz. Transversal section of the central vein of the leaf (A, B). AbE, abaxial epidermis; AdE, adaxial epidermis; FP, fundamental parenchyma; Me, mesophyll; PP, palisade parenchyma; SP, spongy parenchyma; ST, sclerenchyma tissue; Vs, vascular system.

The micromorphological evaluation of a cross section of the leaf sample (Fig. 1A) showed a concave arrangement of the central midrib, which was more pronounced in the lower part with a slight protuberance. The mesophyll showed a well-defined adaxial and abaxial uniseriate epidermis with a fine cuticle on the lateral sides of the central vein. Below the adaxial epidermis, we observed a palisade parenchyma forming two or three continuous layers of elongated cells. The spongy parenchyma exhibited cells of variable size that bordered the abaxial epidermis. An enlargement of the central vein (Fig. 1B) showed the fundamental parenchyma, which was formed by many isometric cells. The sclerenchyma tissue was characterized by lignified thickened walled cells surrounding a well-defined vascular system (xylem and phloem) near the middle of the central vein, which harbored six vascular bundles of variable size.

**Figure 2:**
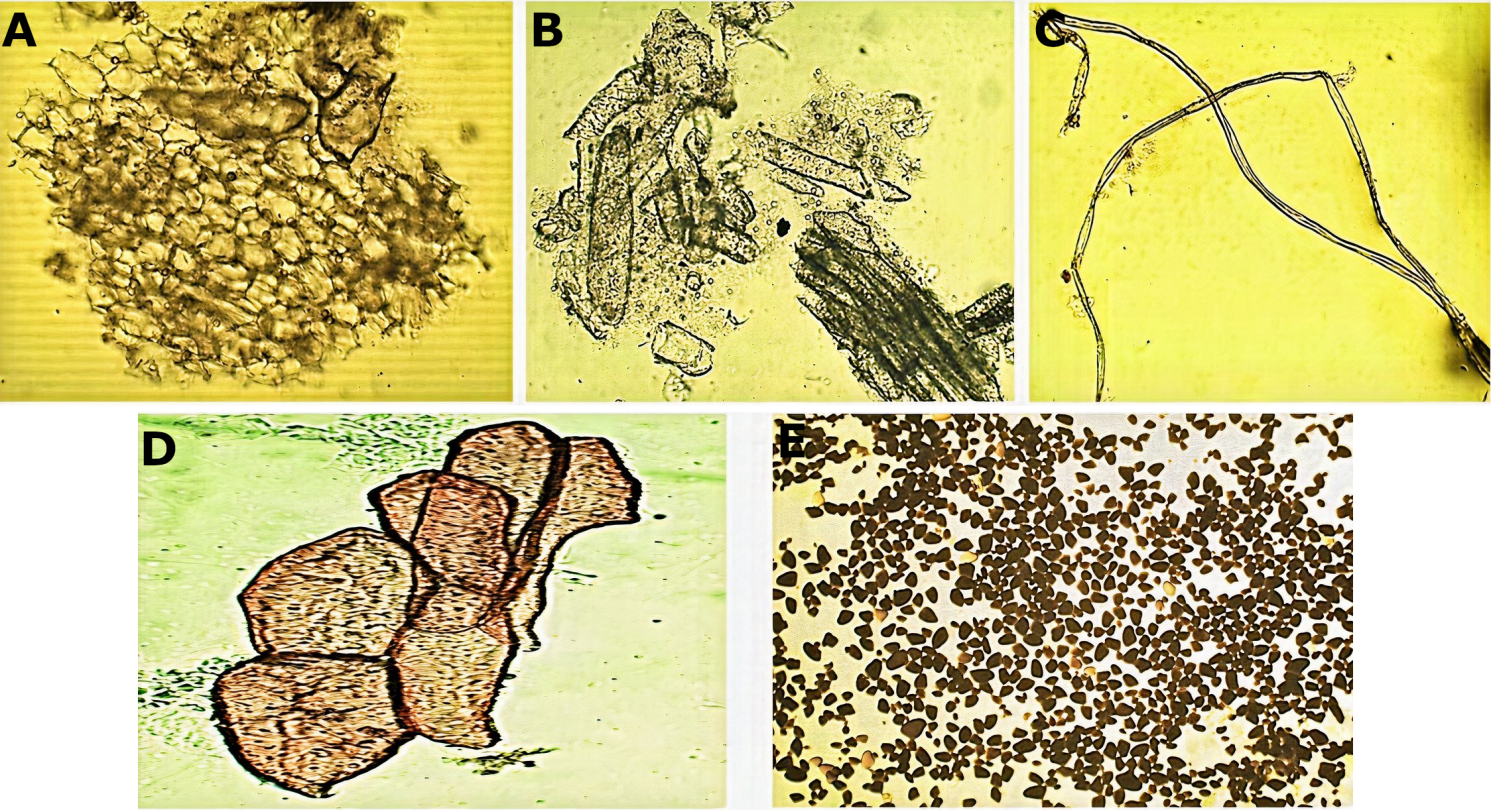
Microscopic characteristics of the powder drug from *Smilax purhampuy* Ruiz rhizome. (A) Parenchyma cells. (B) Xylematic veseels and other elements of the vascular system. (C) Fibers (filiform sclerides). (D) Xylematic veseels, (E) starch granules.

Micromorphological analysis of the drug obtained from the rhizome (Fig. 2) allowed the visualization of a group of cells of the parenchyma of variable size (Fig. 2A) and revealed thickened xylematic vessels associated with other elements of the vascular system (Fig. 2B). Elongated, fusiform, and pointed structures were also visualized, which corresponded to fibers and may suggest a type of filiform sclerides (Fig. 2C). We observed xylematic thickening vessels with holes in another sample of the powder drug (Fig. 2D). Numerous starch granules of variable size were observed showing a blackish coloration with the Lugol reagent (Fig. 2E).

### Molecular barcodes for *Smilax purhampuy* Ruiz plants

Specific PCR amplification was detected for all barcodes except for *trnH-psbA* (data not shown). BLAST analysis was performed for each barcode sequence (Data S1 and S2) and the best hit for all samples for each barcode indicated the *Smilax* species (Table 1). BLAST analysis indicated the presence of *Smilax* spp. using the sequences available at the nr database, including plastid genomes and single locus sequence. The best hits in BLASTn for the different species included: *S. nipponica* for *psbK-psbI* (96.57%, 96.56%, and 93.83% of identity for the three biological replicates, respectively); *S. nipponica* and *S. china* for *rpoB* (99.73% for the three biological replicates); *S. nipponica*, *S. china,* and *S. aspera* for *rpoC1* (100%); *S. sieboldii* f. *inermis* for *atpF-atpH* (90.66%, 90.69%, and 96.47%), *S. fluminensis* (99.65%, 100%), *S. bona-nox* (99.88%) and *S. laurifolia* (99.88%) for *matK*; *S. aspera* (99.82%, 99.82%) and *S. laurifolia* (99.82%) for *rbcL*; and *S. excelsa* (80.49%, 80.05%) for ITS2 (only two biological replicates were sequenced successfully for ITS2).

**Table 1:** Blastn analysis for seven different barcodes of *Smilax purhampuy* Ruiz plants (CIBE-010, CIBE-011, CIBE-012). Results were ranked for the first three with the highest percentage of identity. Species with the best results are bold for each barcode.

Barcode	Code	Blastn rank								
		1			2			3		
		Organism	Accesion	% identity	Organism	Accesion	% identity	Organism	Accesion	% identity
*psbK-psbI*	CIBE-010	***Smilax nipponica***	MT261170.1^a^	96.57%	*Smilax china*	MT261168.1^a^	95.91%	*Smilax glycophylla*	MT261169.1^a^	92.69%
	CIBE-011	***Smilax nipponica***	MT261170.1^a^	96.56%	*Smilax china*	MT261168.1^a^	95.91%	*Smilax glycophylla*	MT261169.1^a^	92.67%
	CIBE-012	***Smilax nipponica***	MT261170.1^a^	93.83%	*Smilax china*	MT261168.1^a^	93.20%	*Smilax glycophylla*	MT261169.1^a^	89.32%
*rpoB*	CIBE-010	***Smilax nipponica***	MT261170.1^a^	99.73%	*Smilax glycophylla*	MT261169.1^a^	99.73%	*Smilax china*	MT261168.1^a^	99.47%
	CIBE-011	***Smilax nipponica***	MT261170.1^a^	99.73%	*Smilax glycophylla*	MT261169.1^a^	99.73%	*Smilax china*	MT261168.1^a^	99.46%
	CIBE-012	***Smilax nipponica***	MT261170.1^a^	99.73%	*Smilax glycophylla*	MT261169.1^a^	99.73%	*Smilax china*	MT261168.1^a^	99.46%
*rpoC1*	CIBE-010	***Smilax nipponica***	MT261170.1^a^	100.00%	*Smilax china*	MT261168.1^a^	100.00%	*Smilax aspera*	EU531650.1	100.00%
	CIBE-011	***Smilax nipponica***	MT261170.1^a^	100.00%	*Smilax china*	MT261168.1^a^	100.00%	*Smilax aspera*	EU531650.1	100.00%
	CIBE-012	***Smilax nipponica***	MT261170.1^a^	100.00%	*Smilax china*	MT261168.1^a^	100.00%	*Smilax aspera*	EU531650.1	100.00%
*atpF-atpH*	CIBE-010	***Smilax sieboldii*f.*inermis***	JN417282.1	90.66%	*Smilax sieboldii*	JN417281.1	90.64%	*Hemidesmus indicus*	NC_047471.1	89.84%
	CIBE-011	***Smilax sieboldii*f.*inermis***	JN417282.1	90.69%	*Smilax sieboldii*	JN417281.1	90.68%	*Smilax glycophylla*	MT261169.1^a^	89.03%
	CIBE-012	***Smilax sieboldii*f.*inermis***	JN417282.1	96.47%	*Smilax sieboldii*	JN417281.1	96.31%	*Smilax china*	MT261168.1^a^	94.69%
*matK*	CIBE-010	***Smilax fluminensis***	JF461414.1	99.65%	*Smilax coriacea*	KJ719950.1	98.72%	*Smilax havanensis*	KF782873.1	98.72%
	CIBE-011	***Smilax fluminensis***	JF461414.1	100.00%	*Smilax coriacea*	KJ719950.1	99.05%	*Smilax havanensis*	KF782873.1	99.05%
	CIBE-012	***Smilax bona-nox***	KC511353.1	99.88%	*Smilax laurifolia*	JF461393.1	99.88%	*Smilax ligneoriparia*	KX432989.1	99.76%
*rbcL*	CIBE-010	***Smilax aspera***	KX394660.1	99.82%	*Smilax aspera*	KX394659.1	99.82%	*Smilax aspera*	KM609079.1	99.82%
	CIBE-011	***Smilax aspera***	KX394660.1	99.82%	*Smilax aspera*	KX394659.1	99.82%	*Smilax aspera*	KM609079.1	99.82%
	CIBE-012	***Smilax laurifolia***	JF944386.1	99.82%	*Smilax china*	MT261168.1^a^	99.45%	*Smilax gaudichaudiana*	KX394669.1	99.45%
ITS2	CIBE-010	***Smilax excelsa***	JF461354.1	80.49%	*Smilax aspera*	KJ719926.1	80.24%	*Smilax aspera*	KJ719924.1	80.24%
	CIBE-011	***Smilax excelsa***	JF461354.1	80.05%	*Smilax aspera*	KJ719926.1	79.81%	*Smilax aspera*	KJ719924.1	79.81

**Notes.**

a Accession numbers refer to plastid complete genome.

**Figure 3:**
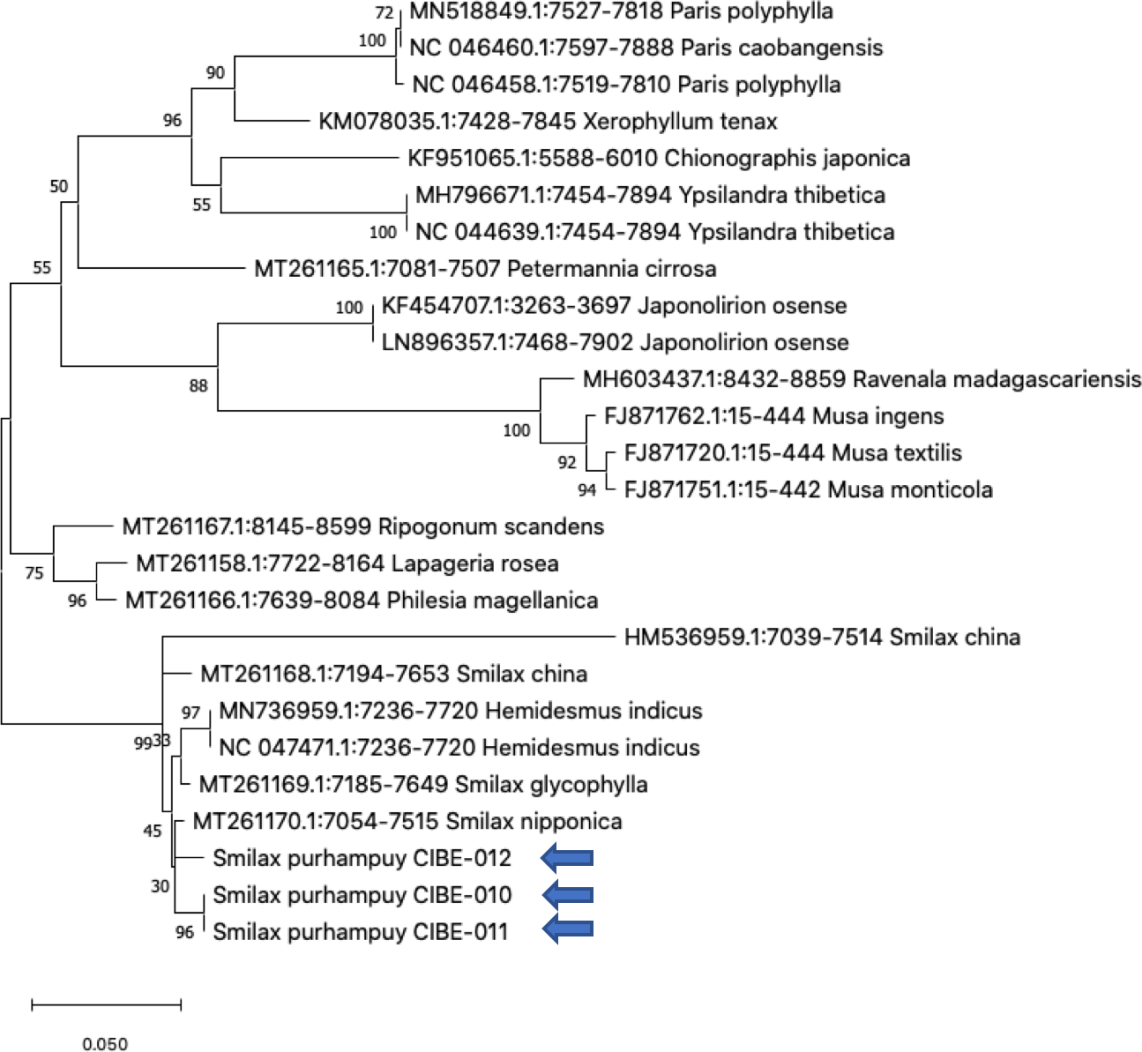
Phylogenetic tree of the *psbK-psbI* spacer with accessions from the genus *Smilax* and different genera selected from the blastn results. Three species from the genus *Paris* was used as outgroup. Maximum Likelihood method based on Tamura 3-parameter model. Boostrap test with 1,000 replicates was performed. The tree with the highest log likelihood (−1728.93) is shown. The percentage of trees in which the associated taxa clustered together is shown next to the branches. Initial tree(s) for the heuristic search were obtained automatically by applying Neighbor-Join and BioNJ algorithms to a matrix of pairwise distances estimated using the Tamura 3 parameter model, and then selecting the topology with superior log likelihood value. A discrete Gamma distribution was used to model evolutionary rate differences among sites (five categories (+*G*, parameter = 1.6020)). The tree is drawn to scale, with branch lengths measured in the number of substitutions per site. This analysis involved 26 nucleotide sequences. There were a total of 368 positions in the final dataset. Evolutionary analyses were conducted in MEGA X (Kumar et al., 2018; Stecher, Tamura & Kumar, 2020). Blue arrows indicate *Smilax purhampuy* Ruiz from Ecuador.

**Figure 4:**
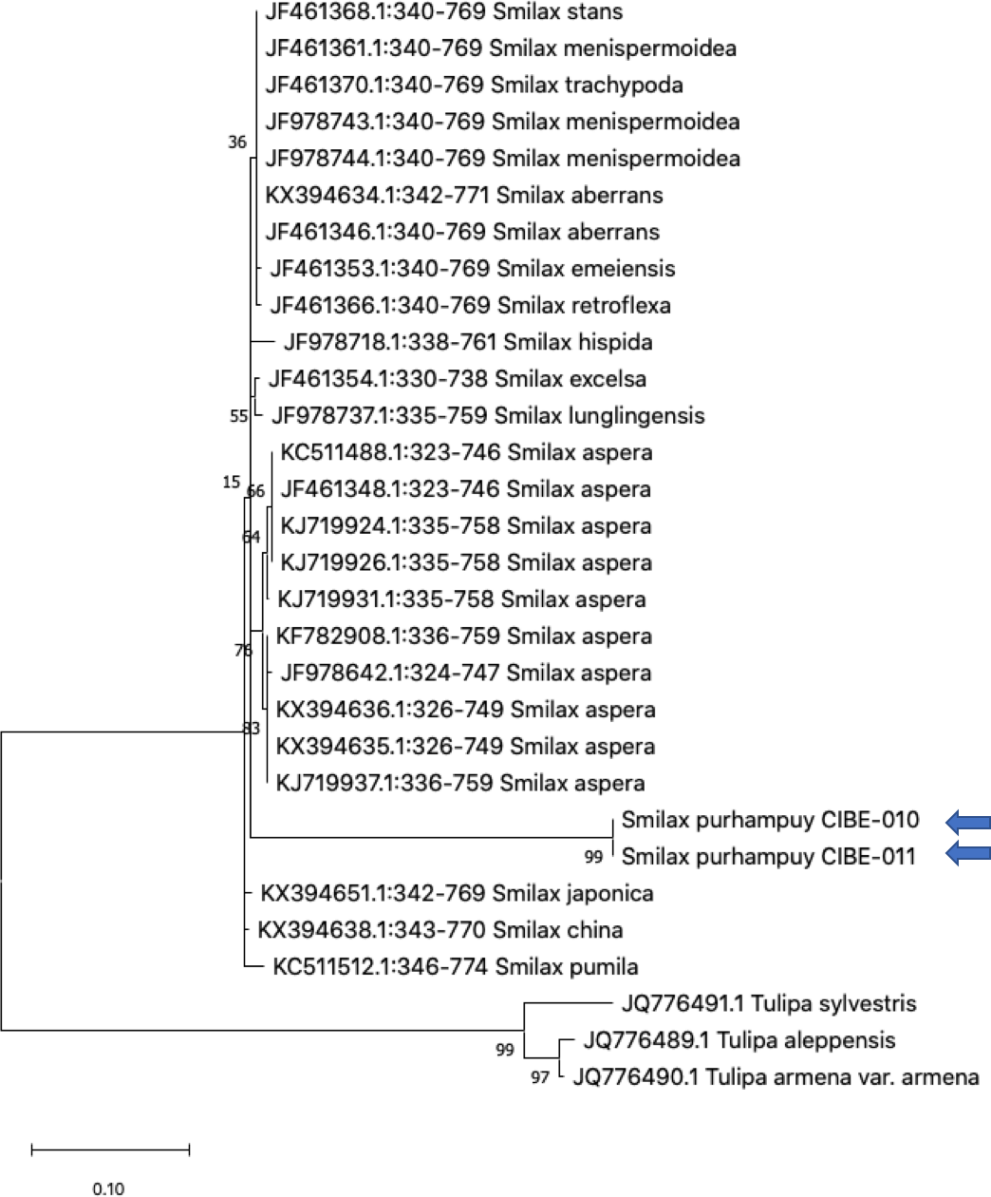
Phylogenetic tree of the ITS2 with accessions from the genus *Smilax* and different genera selected from the blastn results. Three species from the genus *Tulipa* was used as outgroup. Maximum Likelihood method based on Tamura 3-parameter model. The tree with the highest log likelihood (−1405.71) is shown. The percentage of trees in which the associated taxa clustered together is shown next to the branches. Initial tree(s) for the heuristic search were obtained automatically by applying Neighbor-Join and BioNJ algorithms to a matrix of pairwise distances estimated using the Tamura 3 parameter model, and then selecting the topology with superior log likelihood value. A discrete Gamma distribution was used to model evolutionary rate differences among sites (five categories (+*G*, parameter = 0.8022)). The tree is drawn to scale, with branch lengths measured in the number of substitutions per site. This analysis involved 30 nucleotide sequences. There were a total of 436 positions in the final dataset. Evolutionary analyses were conducted in MEGA X (Kumar et al., 2018; Stecher, Tamura & Kumar, 2020). Blue arrows indicate *Smilax purhampuy* Ruiz from Ecuador.

We determined the best models for phylogenetic analysis were: T92+G+I (*rbcL*), T92+G (*matK*, *ITS2*, *psbK-psbI, atpF-atpH*), T92 (*rpoB*), and JC *(rpoC1*) after alignment of the barcode sequences between the *S. purhampuy* from this study and different accessions including other genera. Phylogenetic analysis revealed that for *psbK-psbI*, the *S. purhampuy* sequences shared a clade (99 bootstrap) with different *Smilax* species, including *S. china*, *S. nipponica*, and *S. glycophylla*; and with two accessions from another genus, including *Hemidesmus indicus* (Fig. 3). However, the *S. purhampuy* sequences CIBE-010 and CIBE-011 are grouped in a subclade (bootstrap 96), while *S. purhampuy* CIBE-012 shared a clade with both *S. purhampuy* (bootstrap 30). The other major clades in the phylogenetic tree corresponded to other genera. For the *rpoB* the phylogenetic tree revealed that the *S. purhampuy* samples were in a clade (98 bootstrap) with other *Smilax* species; while other genera including *Brahea* spp., *Philesia magellanica*, *Tricyrtis macropoda*, *Fritillaria* spp., and *Lilium* spp. were in other clades (Fig. S1). The *S. purhampuy* samples for the *rpoC1* were in a clade with different species of *Smilax*, including *S. aspera* (accession number EU531650), *S. nipponica* (MT261170), *S. china* (MT261168), and *S. herbacea* (HQ594138, HQ594139, Fig. S1). The *atpF-atpH* phylogenetic analysis revealed that several *Smilax* species were in the same clade. However, the *S. purhampuy* samples from this study are in a subclade (bootstrap 99). For the *matK* and *rbcL* barcodes, the same pattern was observed where a clade sharing different Smilax species was encountered (Fig. S1), although a subclade was formed with the two *S. purhampuy* samples (CIBE-010, CIBE-011) and *S. fluminensis*; while for the *S. purhampuy* CIBE-012 sample, a branch was shared with *S. aspera* for *matK*. Furthermore, in the *rbcL* phylogenetic tree, a subclade was formed for *S. purhampuy* samples CIBE-010 and CIBE-011 with other species including *S. aspera*, while for the *S. purhampuy* CIBE-012 a clade was shared with *S. domingensis* and *S. lauriflora*. The ITS2 phylogenetic tree (Fig. 4) revealed that the two *S. purhampuy* samples (CIBE-010 and CIBE-011) were in a different clade (bootstrap 99) apart from the other Smilax species, including *S. aspera*, *S. stans*, *S. menispermoidea*, *S. trachypoda*, *S. aberrans*, *S. retroflexa*, *S. excelsa*, *S. lunglingensis*, *S. hispida*, *S. japonica*, *S. china*, and *S. pumila*.

## Discussion

### Microscopic analysis

We made a detailed assessment of the herbal drugs and used microscopy to identify them based on their known histological characteristics (Shailesh et al., 2015). Micromorphological studies are essential for the quality control of plant-derived drugs, since significant details are used to correctly identify the plant and possible adulterants.

The cross section of the *S. purhampuy* leaf indicated that the abaxial and adaxial epidermis are uniseriate, with the existence of an easily perceptible cuticle. Morphoanatomic studies performed on the leaves of various *Smilax* species (*S. brasiliensis*, *S. campestris*, *S. cisoides*, *S. fluminensis*, *S. goyazana*, *S. oblongifolia,* and *S. rufescens*) revealed a non-stratified epidermis with thick cuticle (Martins et al., 2013b). These results correspond to those of previous studies and may be a distinctive anatomical characteristic of the genus.

In most plants, the leaf mesophyll harbors palisade and spongy tissue, which differ in location, cell morphology, and function. In plants with a dorsiventral mesophyll, the palisade tissue is located on the adaxial side and the spongy tissue on the abaxial side; this distribution makes a greater contribution to the photosynthesis process (Yahia et al., 2019). Studies by various researchers have shown that the mesophyll could vary from one leaf to another of the same individual or of different individuals, depending on light intensity and salt concentration (Gapińska & Glińska, 2014). In *S. purhampuy* a dorsiventral mesophyll was observed where the palisade parenchyma is toward the adaxial side and the spongy parenchyma was located toward the abaxial side, which supports the results of Martins & Appezzato-da Glória (2006) for *S. polyantha* and Martins et al. (2013b) for *S. brasiliensis*, *S. campestris*, *S. cissoids*, *S. goyazana*, *S. oblongifolia* and *S. rufescens*. The presence of six vascular bundles of variable size at the level of the central vein of the leaf was unique and differed from other *Smilax* species (*S. brasiliensis*, *S. cissoides*, *and S. fluminensis*), which present three vascular bundles (Martins et al., 2013b).

The presence of parenchyma tissue, xylematic vessels, fibers and starch granules are reported in *S. domingensis* from Cuba and Guatemala (Cáceres et al., 2012; González et al., 2017), which was supported by the present study. The difference lies within the morphology and arrangement of these structures. For example, thickened scalariformly xilematic vessels and fibrotraqueids (fibers) were detected in *S. domingensis*, while thickened xilematic vessel with holes and filiform sclerides (fibers) were observed in the studied species.

### Molecular barcode sequences

Genetic analysis has proven to be an important tool in the standardization of medicinal plants. The genotypic characterization of plant species is important since most plants may show considerable variation in morphology, although they belong to the same genus and species. DNA analysis is useful for the identification of cells, individuals, or species and could help distinguish genuine from adulterated drugs (Kumari & Kotecha, 2016). Different methods may be applied for genotyping in plants. Microsatellite (Martins et al., 2013a; Ru et al., 2017; Qi et al., 2017) and DNA barcodes (Cameron & Fu, 2006; Qi et al., 2013) have been used for genotyping *Smilax* species and have shown phylogenetic relationships between different *Smilax* species. Additionally, Sulistyaningsih et al. (2018) used the DNA barcode *rbcL* for phylogenetic analysis of *Smilax* spp. in Java, Indonesia, concluding that *rbcL* could be used to identify at the genus but not at species level.

The analysis of DNA barcodes could be used as a complementary analysis for the identification of plants species, especially when *Smilax* species show considerable phenotypic variations within populations (Cameron & Fu, 2006). The recommended barcodes for species identification are 2-*locus rbcL* and *matK* (CBOL Plant Working Group, 2009). Generally, the BLASTn analysis relies in the presence of those species in the GenBank for species identification; consequently, the results presented in the BLAST analysis and in the phylogenetic trees depended on the sequences available in the nr database in the GenBank. Therefore, a complete analysis using different species of *Smilax* should be performed in the future for all the DNA barcodes tested.

We analyzed the three samples taxonomically identified as *S. purhampuy* Ruiz (CIBE-010, CIBE-011, CIBE-012) which were more similar than other *Smilax* species, including *S. nipponica*, *S. glycophylla*, *S. herbacea*, *S. china*, *S. sieboldin*, *S. aspera*, *S. stans*, *S. menispermoidea*, *S. trachypoda*, *S. aberrans,* and *S. pumila*. These results were also observed in the phylogenetic trees for *psbK-psbI* spacer, *atpF-atpH* spacer, and ITS2. However, few accessions were encountered in the GenBank for the DNA barcodes *psbK-psbI* spacer and *atpF-atpH* spacer. There should be additional study of the different *Smilax* species for these barcodes; however, other DNA barcodes may accurately identify the genus level. The ITS2 revealed a low percentage of identification (80.49%) with BLASTn, suggesting that species differentiation could be detected using the ITS2. The results from other studies have indicated a better resolution for species identification using the ITS2 in medicinal plants (Techen et al., 2014; Zhang et al., 2016; Bustamante et al., 2019; Sarmiento-Tomalá et al., 2020).

Our results determined that the *rpoC1* sequence was not accurate at the species level, and that *matK* could not be used to discriminate between *S. purhampuy* and *S. fluminensis*. Furthermore, the *rbcL* barcode could not be used for species differentiation in the *Smilax* genus, as a low bootstrap value was observed in the different clades formed. The *psbK-psbI*, *atpF-atpH,* and *ITS2* had a better resolution at the species level for *S. purhampuy*. Future research should include the sequencing of selected barcodes (*rbcL*, *matK*, *psbK-psbI* spacer, *atpF-atpH* spacer, and ITS2) for different species of the *Smilax* genus found in Ecuador with biological replicates. Further studies should establish a reliable DNA barcode analysis and test different 2-*locus* combinations to determine which barcode should be used for species identification in the *Smilax* genus.

## Conclusions

We determined the morphological characteristics and conducted molecular barcode analysis on *S. purhampuy* Ruiz plants collected in Ecuador. The micromorphological characteristics of the leaves and rhizomes were described for the first time, which constitutes a novel contribution to the botanical characterization of the species. The taxonomic classification of *Smilax* was confirmed by the molecular barcodes used, including *psbK-psbI, rpoB, rpoC, atpF-atpH, mat*K, *rbc*L, and ITS2. Furthermore, the barcodes sequences *psbK-psbI, atpF-atpH,* and ITS2 indicated a better resolution at the species level than the other barcodes tested in this study. These barcodes (*psbK-psbI, atpF-atpH,* and ITS2) could be used to identify other species in the genus *Smilax*. However, further molecular barcode analysis should be performed on *Smilax* spp. from Ecuador to determine its diversity and to complete its taxonomic classification. Furthermore, the medicinal properties of the *Smilax* plants used in this study should be studied in greater detail.

##  Supplemental Information

10.7717/peerj.11028/supp-1 Figure S1 Phylogenetic analysis of molecular barcode sequences

10.7717/peerj.11028/supp-2 Table S1 Primers used for amplification of the barcodes *psbA-trnH* spacer*, psbK-psbI* spacer*, rpoB, rpoC1, atpF-atpH* spacer*, rbc* L, *mat* K, and ITS2

10.7717/peerj.11028/supp-3 Data S1 Barcode DNA sequences of *Smilax purhampuy* Ruiz

10.7717/peerj.11028/supp-4 Data S2 Accession numbers of *Smilax purhampuy* Ruiz (GUAY 13117) barcode sequences at GenBank

## Additional Information and Declarations

### Field Study Permissions

The following information was supplied relating to field study approvals (i.e., approving body and any reference numbers): Samples were collected for identification in the framework of the GUAY Herbarium, Facultad de Ciencias Naturales, Universidad de Guayaquil.

### Data Availability

The following information was supplied regarding data availability: Sequences are available in the Supplemental Files and at GenBank: MT740231, MT740232, MT740233, MT740234, MT740235, MT740236, MT740237, MT740238, MT740239, MT740240, MT740241, MT740242, MT734663, MT734664, MW300280, MW300281, MW300282, MW300277, MW300278, MW300279.
