# Supplementary material for: Molecular barcode and morphological analysis of Smilax purhampuy Ruiz, Ecuador
Source: PeerJ. 2021 Mar 18;9:e11028. doi: 10.7717/peerj.11028 (PMC7982074; doi:10.7717/peerj.11028)
Supplement: Figure S1 [file peerj-09-11028-s001.docx]

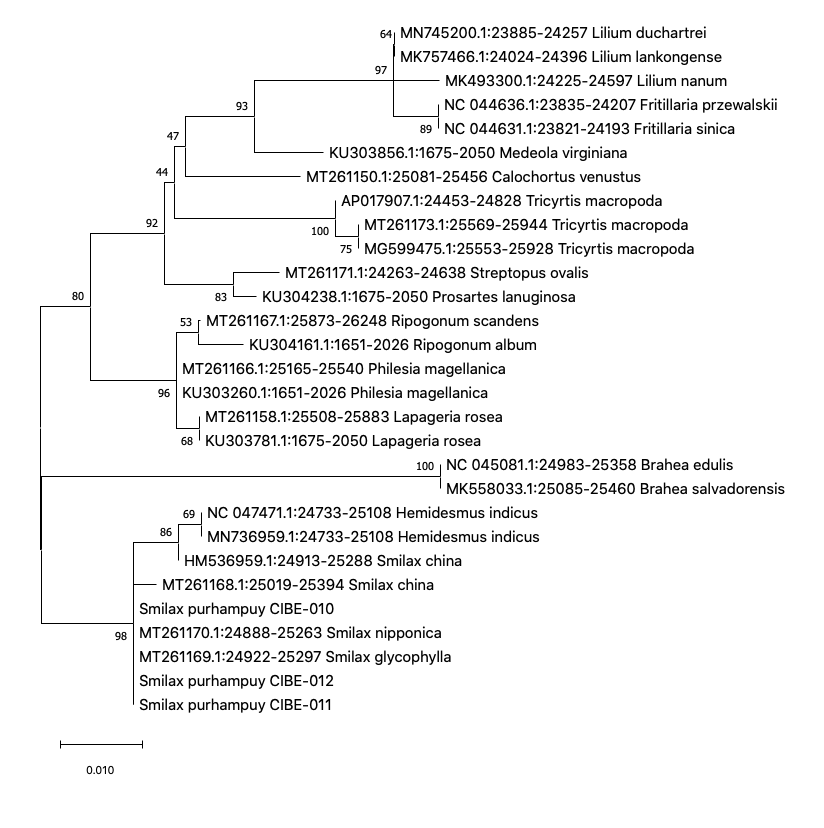


Phylogenetic tree of the *rpoB* with accessions from the genus *Smilax* and different genera selected from the blastn results. Maximum Likelihood method based on Tamura 3-parameter model. The tree with the highest log likelihood (-940.74) is shown. The percentage of trees in which the associated taxa clustered together is shown next to the branches. Initial tree(s) for the heuristic search were obtained automatically by applying Neighbor-Join and BioNJ algorithms to a matrix of pairwise distances estimated using the Tamura 3 parameter model, and then selecting the topology with superior log likelihood value. The tree is drawn to scale, with branch lengths measured in the number of substitutions per site. This analysis involved 29 nucleotide sequences. Codon positions included were 1st+2nd+3rd+Noncoding. There were a total of 364 positions in the final dataset. Evolutionary analyses were conducted in MEGA X (Kumar et al., 2018; Stecher et al., 2020). Blue arrows indicate *Smilax* *purhampuy* Ruiz from Ecuador.


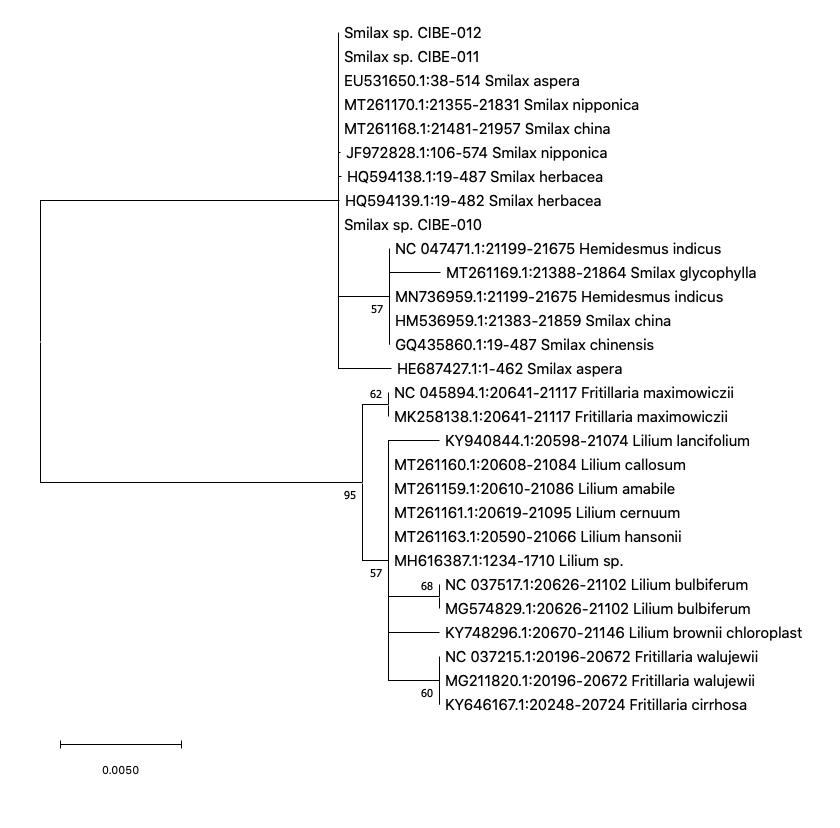


Phylogenetic tree of the *rpoC1* with accessions from the genus *Smilax* and different genera selected from the blastn results. Maximum Likelihood method based on Jukes-Cantor model. The tree with the highest log likelihood (-751.95) is shown. The percentage of trees in which the associated taxa clustered together is shown next to the branches. Initial tree(s) for the heuristic search were obtained automatically by applying Neighbor-Join and BioNJ algorithms to a matrix of pairwise distances estimated using the Jukes-Cantor model, and then selecting the topology with superior log likelihood value. The tree is drawn to scale, with branch lengths measured in the number of substitutions per site. This analysis involved 29 nucleotide sequences. Codon positions included were 1st+2nd+3rd+Noncoding. There were a total of 449 positions in the final dataset. Evolutionary analyses were conducted in MEGA X (Kumar et al., 2018; Stecher et al., 2020). Blue arrows indicate *Smilax* *purhampuy* Ruiz from Ecuador.


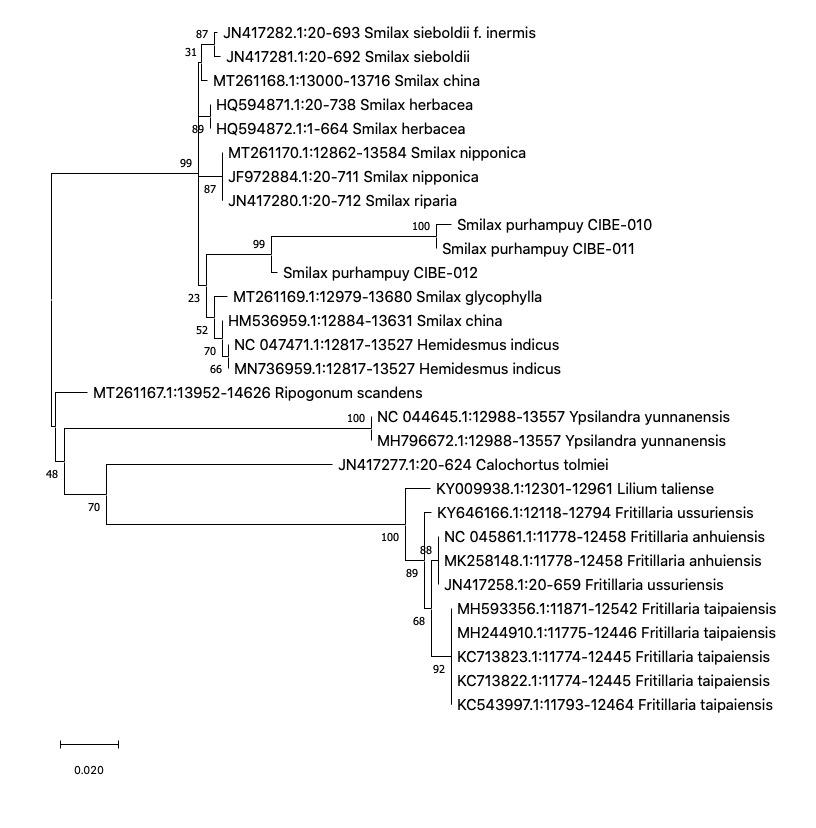


Phylogenetic tree of the *atpF-atpH* spacer with accessions from the genus *Smilax* and different *ge*n*era* selected from the blastn results. Maximum Likelihood method based on Tamura 3-parameter model. The tree with the highest log likelihood (-1811.61) is shown. The percentage of trees in which the associated taxa clustered together is shown next to the branches. Initial tree(s) for the heuristic search were obtained automatically by applying Neighbor-Join and BioNJ algorithms to a matrix of pairwise distances estimated using the Tamura 3 parameter model, and then selecting the topology with superior log likelihood value. A discrete Gamma distribution was used to model evolutionary rate differences among sites (5 categories (+*G*, parameter = 0.9507)). The tree is drawn to scale, with branch lengths measured in the number of substitutions per site. This analysis involved 29 nucleotide sequences. There were a total of 557 positions in the final dataset. Evolutionary analyses were conducted in MEGA X (Kumar et al., 2018; Stecher et al., 2020). Blue arrows indicate Smi*l*ax *purhampuy* Ruiz from Ecuador.


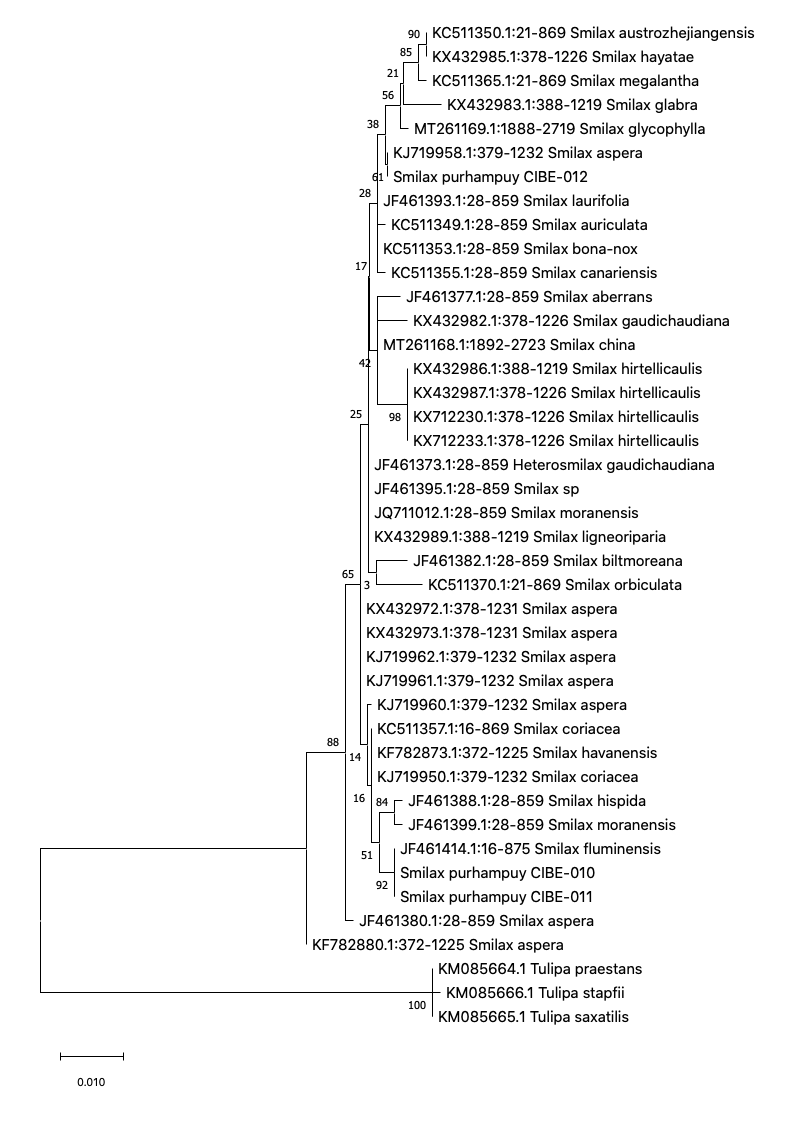


Phylogenetic tree of the *matK* with accessions from the genus *Smilax* and different genera selected from the blastn results. Three species from the *ge*n*us* *Tulipa* was used as outgroup. Maximum Likelihood method based on Tamura 3-parameter model. The tree with the highest log likelihood (-1835.37) is shown. The percentage of trees in which the associated taxa clustered together is shown next to the branches. Initial tree(s) for the heuristic search were obtained automatically by applying Neighbor-Join and BioNJ algorithms to a matrix of pairwise distances estimated using the Tamura 3 parameter model, and then selecting the topology with superior log likelihood value. A discrete Gamma distribution was used to model evolutionary rate differences among sites (5 categories (+*G*, parameter = 0.4762)). The tree is drawn to scale, with branch lengths measured in the number of substitutions per site. This analysis involved 42 nucleotide sequences. Codon positions included were 1st+2nd+3rd+Noncoding. There were a total of 838 positions in the final dataset. Evolutionary analyses were conducted in MEGA [ (Kumar et al., 2018; Stecher et al., 2020). Blue arrows indicate *Smilax* *purhampuy* Ruiz from Ecuador.


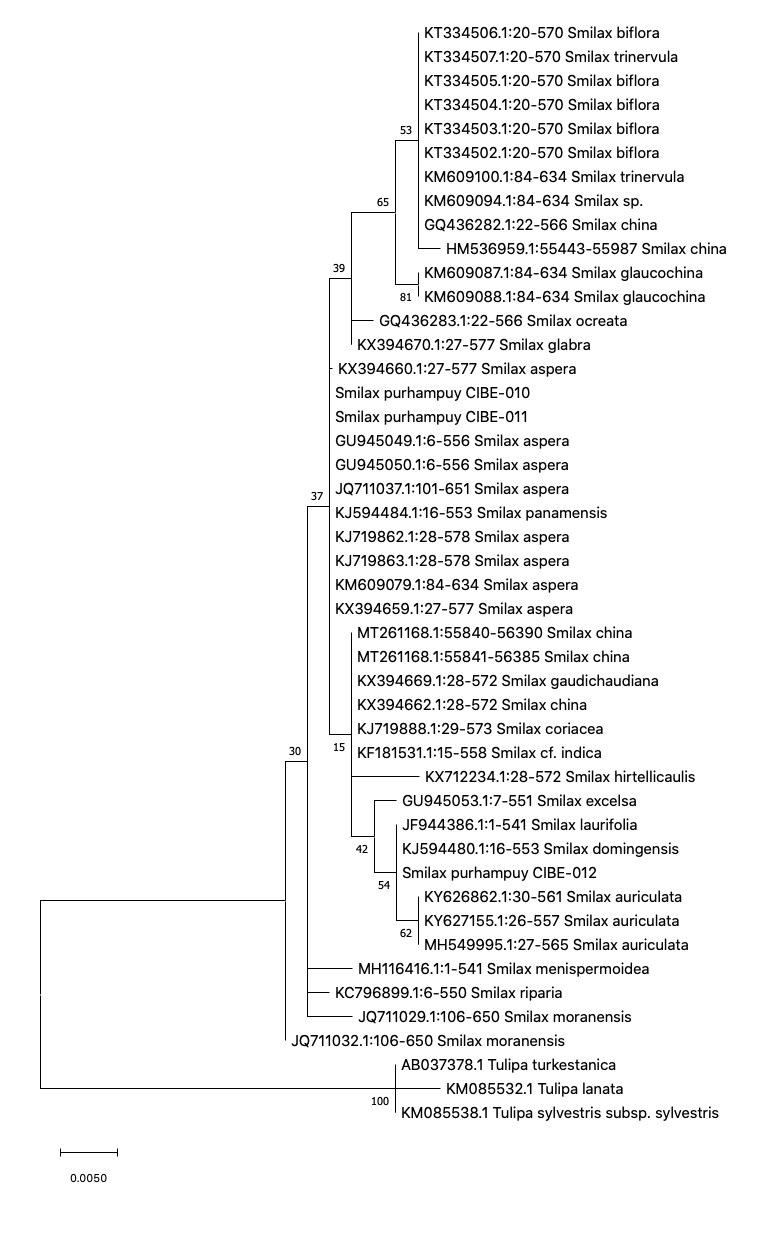


Phylogenetic tree of the *rbc*L with accessions from the genus *Smilax* and different genera selected from the blastn results. Three species from the genus *Tulipa* was used as outgroup. Maximum Likelihood method based on Tamura 3-parameter model. The tree with the highest log likelihood (-994.51) is shown. The percentage of trees in which the associated taxa clustered together is shown next to the branches. Initial tree(s) for the heuristic search were obtained automatically by applying Neighbor-Join and BioNJ algorithms to a matrix of pairwise distances estimated using the Tamura 3 parameter model, and then selecting the topology with superior log likelihood value. A discrete Gamma distribution was used to model evolutionary rate differences among sites (5 categories (+*G*, parameter = 0.0626)). The rate variation model allowed for some sites to be evolutionarily invariable ([+*I*], 49.72% sites). The tree is drawn to scale, with branch lengths measured in the number of substitutions per site. This analysis involved 46 nucleotide sequences. Codon positions included were 1st+2nd+3rd+Noncoding. There were a total of 528 positions in the final dataset. Evolutionary analyses were conducted in MEGA X (Kumar et al. 2018; Stecher et al., 2020). Blue arrows indicate *Smilax* *purhampuy* Ruiz from Ecuador.
