# Supplementary material for: Molecular barcode and morphological analysis of Smilax purhampuy Ruiz, Ecuador
Source: PeerJ. 2021 Mar 18;9:e11028. doi: 10.7717/peerj.11028 (PMC7982074; doi:10.7717/peerj.11028)
Supplement: Table S1 [file peerj-09-11028-s002.docx]

Table S1. Primers used for amplification of the barcodes *psbA-trnH* spacer*, psbK-psbI* spacer*, rpoB, rpoC1, atpF-atpH* spacer*, rbc*L, *mat*K, and ITS2

| **Primer pairs** | **Sequence** | ***Locus*** | **Annealing temperature** | **Reference** |
| --- | --- | --- | --- | --- |
| trnHf_05 | CGCGCATGGTGGATTCACAATCC | *psbA-trnH* | 60°C | Basak et al. (2019) |
| psbA3_f | GTTATGCATGAACGTAATGCTC |  |  |  |
| psbK_F | TTAGCCTTTGTTTGGCAAG | *psbK-psbI* | 56°C | Basak et al. (2019) |
| psbI_R | AGAGTTTGAGAGTAAGCAT |  |  |  |
| rpoB_2F | ATGCAACGTCAAGCAGTTCC | *rpoB* | 50°C | Basak et al. (2019) |
| rpoB_3R | CCGTATGTGAAAAGAAGTATA |  |  |  |
| rpoC1_2F | GGCAAAGAGGGAAGATTTCG | *rpoC1* | 60°C | Basak et al. (2019) |
| rpoC1_4R | CCATAAGCATATCTTGAGTTGG |  |  |  |
| atpF_F | ACTCGCACACACTCCCTTTCC | *atpF-atpH* | 56°C | Basak et al. (2019) |
| atpH_R | GCTTTTATGGAAGCTTTAACAAT |  |  |  |
| rbcLA_F | ATGTCACCACAAACAGAGACTAAAGC | *rbc*L | 60°C | Costion et al. (2011) |
| rbcLA_R | GTAAAATCAAGTCCACCRCG |  |  |  |
| matK_3F_KIMF | CGTACAGTACTTTTGTGTTTACGAG | *mat*K | 56°C | Costion et al. (2011) |
| matK_1R_KIMR | ACCCAGTCCATCTGGAAATCTTGGTTC |  |  |  |
| S2F | ATGCGATACTTGGTGTGAAT | ITS2 | 60°C | Cheng et al. (2015) |
| S3R | GACGCTTCTCCAGACTACAAT |  |  |  |

**References**

Basak S, Aadi Moolam R, Parida A, Mitra S, Rangan L. 2019. Evaluation of rapid molecular diagnostics for differentiating medicinal Kaempferia species from its adulterants. Plant Diversity 41(3):206–211 DOI 10.1016/j.pld.2019.04.003.

Cheng T, Xu C, Lei L, Li C, Zhang Y, Zhou S. 2015. Barcoding the kingdom Plantae: new PCR primers for ITS regions of plants with improved universality and specificity. Molecular Ecology Resources 16(1):138–149.

Costion C, Ford A, Cross H, Crayn D, Harrington M, Lowe A. 2011. Plant DNA barcodes can accurately estimate species richness in poorly known floras. PLOS ONE 6(11):e26841 DOI 10.1371/journal.pone.0026841.
