# Supplementary material for: Molecular barcode and morphological analysis of Smilax purhampuy Ruiz, Ecuador
Source: PeerJ. 2021 Mar 18;9:e11028. doi: 10.7717/peerj.11028 (PMC7982074; doi:10.7717/peerj.11028)
Supplement: Data S1 [file peerj-09-11028-s003.docx]

Data S1. Barcode DNA sequences of *Smilax purhampuy* Ruiz

> Smilax purhampuy Ruiz, ATPase subunit I (atpF), partial cds; atpF-atpH intergenic spacer; voucher CIBE-010.

AGCACTACACTTAGATTTATTGGATTTGTTGCTAAAATATCGGTATTAAACCCGAAACTACCTGCGTATGGCCAATAGCCCAAGGAAACGAAAAAATAGGTTATATTTTTCATATGCTCTCCTCTTTCTTATAGATAAGACTAACAAGGAACAGAGTTCTTTTTGTATCATCTCGCTCGCCCCTTTTTTGATCGATTTCTTTTTTTTTTTTTTTATTAATTTAATTTTATTTTTTAGGGAAGTAAATTAAATCATCTATTAATTTATAATGAAATTTGAAAATTTTCAAATTTATTATTATTATTTTTTTTTTTTAATTTCCCAAAAAAAAAAAACTTATTAGGTTAGGCCCAAGGTCCCACGTTAATTGCAAARTATCCCGTTTGTTGAARGGCTTCCAAACAAAAAGGTTTTTGTTGTMCTAAGGGGGGRAAGGAAAAAAGCRASCAAAACCCCAAATTCCCCACCCCCAAATCATCCCTTCCCGGGGTWTTGTCCCAAWAAAWAA

>Smilax purhampuy Ruiz, ATPase subunit I (atpF), partial cds; atpF-atpH intergenic spacer,;ATPase subunit III (atpH), partial cds, voucher CIBE-011.

AGCACTACACTTAGATTTATTGGATTTGTTGCTAAAATATCGGTATTAAACCCGAAACTACCTGCGTATGGCCAATAGCCCAAGGAAACGAAAAAATAGGTTATATTTTTCATATGCTCTCCTCTTTCTTATAGATAAGACTAACAAGGAACAGAGTTCTTTTTGTATCATCTCGCTCGCCCCTTTTTTGATCGATTTCTTTTTTTTTTTTTTTATTAATTTAATTTTATTTTTTATGGAAGTAAATTAAATCATCTATTAATTTATAATGAAATTTGAAAATTTTCAAATTTATTATTATTATTTTTTTTTTTTAAKTTCCCAAAAAAAAAAAACTTATWAGGTTAGGCCCWAGGTCCCACGTTAATTGCAAARTATCTCGTTTGTTGAAAGGCTTCCWAACAAAAAGGTTTTTGTTGTACTAAGGGGGGGAAGGAAAAAAGCAAGCAAAWMCCCAAATTCCCCACCCCCAAATCATCCCTTCCCGGGGTATTGTCCCAATAAAAAAGWAATTGTAGRAGYCAAGGGTTGATWTAATTAAAAAAASCAAACGGCAAGCCCAATTTAATTTAATCAAATTTAATTTAAAAAAAWAACTAAAAAAAAAGCSCGWAACSCATTTTTTCACTTTTCTAATTTTAGGATTAATTTAAACAAAAGGATTTGCAAATAAAASCCCTAAGGCTACAACCAGYCCGTAAATTGTAACGYCCC

>Smilax purhampuy Ruiz, ATPase subunit I (atpF), partial cds; atpF-atpH intergenic spacer; ATPase subunit III (atpH), partial cds, voucher CIBE-012.

AGCACTACACTTAGATTTATTGGATTTGTTGCTAAAATATCGGTATTAAACCCGAAACTACCTGCGTATGGCCAATAGCCCAAGGAAACGAAAAAATAGGTTATATTTTTCATATGCTCTCCTCTTTCTTATAGATAAGACTAACAAGGAACAGAGTTCTTTTTGTATCATCTCGCTCGCCCCTTTTTTGATCGATTTCTTTTTTTGTTTTATTAATTGAATTTTATTTTTTATGGAAATAGATTAAATCATCTATTAATTTATAATGAAATTTGAGAATTTTCAAATTTATTATTATTTTTTTTTTTTTTTTAAGTTCTCAATAAAAAAATACTTATTAGGTTAGGTCCTAGGTCTCACGTTAATTGCAAAGTATCTCGTTTGTTGAAAGGCTTCCTAAAAAAAAGGTTTTTGTTGTACTAAGGGGGGGAAGGAAAAAAGCGAGCGGATACCCAAATTCCYCAYCCYCAAATCAGCCCTTCCCGGGGTATTGTCTCAATAAATAAGTAATTGTAGGAGTCAAGTGTTGATATAATTAAAAAAAGCAAACGGCAAGTCCAAGTTAAGTTAATCAAATTTAATTTAATAAAATAACTAAWAAAAAAGCGCGTAACGCAYTTTTTCACATTTCTAATTTTAGGATTAATTTAAACAAAAGGATTTGCAAATAAAAGCGCTAAKGCTACRACCAGTCCGTAAATTG

>Smilax purhampuy Ruiz, psbk-psbI intergenic region, voucher CIBE-010.

TCGATGATCTTTATATTCTCCTAAATTCATTATTTTTTTGATAAAAAAAGATTCTAAAAATTGATAAGATCAGATAAGTCTTATATTATAAACCCTCGATTCAAAAATGGAAATTTTATATATTAAATAACCGTAGCAATGAATGGCATTAAATTTTGATCAATTTATTTCCCTCGTTCTGTTCTGACCTTCCGGTGAAGAAAGACTTTATTAGGTCTTCCACAATACCTAATTGTGGATATAACAATAAAATTTTGATTATGAAGGAATCAGAATCTTATTCCAAAGAAATTCGTTAAAATTACTTTTTTTTTTCAGGAAAACACTGCATTTTTTTCTTTTTGAGACATGTCATGTCAAAATAGCATATGTGGTACAAAAAAAAGAAAGAATAGGTAATCTATTCCCCTTTTCAATTAAAAAAAAATGATCTTATCTTGGAGATTGTGTAATGCTTA

>Smilax purhampuy Ruiz, psbk-psbI intergenic region, voucher CIBE-011.

TTCGATGATCTTTATATTCTCCTAAATTCATTATTTTTTTGATAAAAAAAGATTCTAAAAATTGATAAGATCAGATAAGTCTTATATTATAAACCCTCGATTCAAAAATGGAAATTTTATATATTAAATAACCGTAGCAATGAATGGCATTAAATTTTGATCAATTTATTTCCCTCGTTCTGTTCTGACCTTCCGGTGAAGAAAGACTTTATTAGGTCTTCCACAATACCTAATTGTGGATATAACAATAAAATTTTGATTATGAAGGAATCAGAATCTTATTCCAAAGAAATTCGTTAAAATTACTTTTTTTTTTCAGGAAAACACTGCATTTTTTTCTTTTTGAGACATGTCATGTCAAAATAGCATATGTGGTACAAAAAAAAGAAAGAATAGGTAATCTATTCCCCTTTTCAATTAAAAAAAAATGATCTTATCTTGGAGATTGTGTAATGCT

>Smilax purhampuy Ruiz, psbk-psbI intergenic region, voucher CIBE-012.

TCGATGATCTTTATATTCTCCTAAATTCATTATTTTTTTGATAAAAAAAGATTCTAAAAATTGATAAGATCAGATAAGTCTTATATTATAAACCCTCGATTCAAAAATGGAAATTTTATATATTGAATAACCGTAGCAATGAGTGGCATTAAATTTTGATCAATTTATTTTCCTCGTTCTGTTCTGACCTTCCGGTGAAGAAAGACTTTATTAGGTCTTCCACAATACCTAATTGTGGATATAACAAGAAAATTTTGATTACGAAGGAAAAGGAATCAGAATCTTATTCCAAAGAAATTCGTTAAAATGACTTTTTTTTTCAGGAAAACACTGCATTTTTTTCTTTTTGAGACATGTCATGTCAAAATAGCATATGTGGTACAAAAAAAAGAAAGAATAGGTAATCTATTCCCCTTTTCAATTAAAAAAAAAAAAAAAAAAATGATCTTATCTTGGAAATTGTGTAAGGCTAACCCCCAAAAA

>Smilax purhampuy Ruiz; 5.8S ribosomal RNA gene, partial sequence; internal transcribed spacer 2, complete sequence; and large subunit ribosomal RNA gene, partial sequence; voucher CIBE-010

CCGAGGGCACGCTTGCCTGGGCATCATGCATCACGTCGCTCCGCCTCCCATGGGTGCTAGATGCAGAGATTGGCTCCCCGTGTGTGAGGGCAGCGGGCCCAAGCATGGGCTGCCGGCTTGGATGGGCACGATGCTGACTCCACGCATGCGAACCAAACACCGTGTGCCCCGGCGCTAACAGCATATGGCCCTTCGGAACCTCAGAGGGCTCGCTGGCCCTCCCCAGGGATGGGCGAGATGCCTTGCACCACGACCCAAGTCAGGCAGGGCCAACCGCCGAGTTTAAGGATATCAATAAGCATAGGATAAGAAACTTACGAGGATTCCCCTAGTAACAGCGAGCGAACTGGGATCAACCTAGCTTGAGAATTGGGGGGTCGCTCCCTCTGAATTGTAGTCTGAGAAAAACCGTCAAAA

>Smilax purhampuy Ruiz; 5.8S ribosomal RNA gene, partial sequence; internal transcribed spacer 2, complete sequence; and large subunit ribosomal RNA gene, partial sequence; voucher CIBE-011

CCGAGGGCACGGCTTGCCTGGGCATCATGCATCACGTCGCTCCGCCTCCCATGGGTGCTAGATGCAGAGATTGGCTCCCCGTGTGTGAGGGCAGCGGGCCCAAGCATGGGCTGCCGGCTTGGATGGGCACGATGCTGACTCCACGCATGCGAACCAAACACCGTGTGCCCCGGCGCTAACAGCATATGGCCCTTCGGAACCTCAGAGGGCTCGCTGGCCCTCCCCAGGGATGGGCGAGATGCCTTGCACCACGACCCAAGTCAGGCAGGGCCAACCGCCGAGTTTAAGGATATCAATAAGCATAGGATAAGAAACTTACGAGGATTCCCCTAGTAACAGCGAGCGAACTGGGATCAACCTAGCTTGAGAATTGGGGGGTCGCTCCCTCTGAATTGTA

>Smilax purhampuy Ruiz, ribulose-1,5-bisphosphate carboxylase/oxygenase large subunit (rbcL) gene, partial cds; voucher CIBE-010

AAGATTACAAATTGACTTATTATACTCCTGACTATGAAACCAAAGATACTGATATCTTGGCAGCATTCCGAGTAACTCCTCAACCCGGAGTTCCGCCTGAAGAGGCAGGGGCAGCGGTAGCCGCAGAATCTTCTACTGGTACATGGACAACTGTGTGGACTGATGGACTTACCAGTCTTGATCGTTACAAAGGACGATGCTACCACATAGAGAGCGTTGTTGGGGAGGAAAATCAATATATTGCTTATGTAGCTTATCCTTTAGACCTTTTTGAAGAAGGCTCGGTTACTAACATGTTTACTTCCATTGTGGGTAATGTATTTGGTTTCAAAGCCCTACGAGCTCTACGTCTAGAGGATTTGCGAATTCCTACTTCTTATTCCAAAACTTTCCAAGGCCCACCCCATGGCATCCAAGTTGAAAGAGATAAATTGAACAAGTATGGTCGTCCCCTATTGGGATGTACCATTAAACCAAAATTGGGATTATCCGCAAAGAACTACGGTAGAGCGGTTTATGAATGTCTGCGCGGTGGACTTT

>Smilax purhampuy Ruiz, ribulose-1,5-bisphosphate carboxylase/oxygenase large subunit (rbcL) gene, partial cds; voucher CIBE-011

AAGATTACAAATTGACTTATTATACTCCTGACTATGAAACCAAAGATACTGATATCTTGGCAGCATTCCGAGTAACTCCTCAACCCGGAGTTCCGCCTGAAGAGGCAGGGGCAGCGGTAGCCGCAGAATCTTCTACTGGTACATGGACAACTGTGTGGACTGATGGACTTACCAGTCTTGATCGTTACAAAGGACGATGCTACCACATAGAGAGCGTTGTTGGGGAGGAAAATCAATATATTGCTTATGTAGCTTATCCTTTAGACCTTTTTGAAGAAGGCTCGGTTACTAACATGTTTACTTCCATTGTGGGTAATGTATTTGGTTTCAAAGCCCTACGAGCTCTACGTCTAGAGGATTTGCGAATTCCTACTTCTTATTCCAAAACTTTCCAAGGCCCACCCCATGGCATCCAAGTTGAAAGAGATAAATTGAACAAGTATGGTCGTCCCCTATTGGGATGTACCATTAAACCAAAATTGGGATTATCCGCAAAGAACTACGGTAGAGCGGTTTATGAATGTCTGCGCGGT

>Smilax purhampuy Ruiz, ribulose-1,5-bisphosphate carboxylase/oxygenase large subunit (rbcL) gene, partial cds; voucher CIBE-012

AAGATTACAAATTGACTTATTATACTCCTGACTATGAAACCAAAGATACTGATATCTTGGCAGCATTCCGAGTAACTCCTCAACCCGGAGTTCCGCCTGAAGAGGCAGGGGCAGCGGTAGCCGCAGAATCTTCTACTGGTACATGGACAACTGTGTGGACTGATGGACTTACCAGTCTTGATCGTTACAAAGGACGATGCTACCACATAGAGAGCGTTATTGGGGAGGAAAATCAATATATTGCTTATGTAGCTTATCCTTTAGACCTTTTTGAAGAAGGCTCGGTTACTAACATGTTTACTTCCATTGTGGGTAATGTATTTGGTTTCAAAGCCCTACGAGCTCTACGTCTGGAGGATTTGCGAATTCCTACTTCTTATTCCAAAACTTTCCAAGGCCCACCCCATGGCATCCAAGTTGAAAGAGATAAATTGAACAAGTATGGTCGTCCCCTATTGGGATGTACAATTAAACCAAAATTGGGATTATCCGCAAAGAACTACGGTAGAGCGGTTTATGAATGTCTGCGCGGTGA

>Smilax purhampuy Ruiz, maturase K (matK) gene, partial cds, voucher CIBE-010

TCTTGGTTCAAATCCTTCAATGCTGGATTCAAGATGTTCCCTCTTTACATTTATTGCGACTCTTTCTTCATAAATATCATAATTTGAATAGATTTATTACTCTGAATAAATCTATTTACGTTTTTTCAAAAGAAAATACAAGACTATTTCGCTTCCTGTATAATTCTTATGTAGCTGAATGTGAATTTTTATTTGTTTTTCTTCGTAAACAATCCTATTATTTACGATCAACATCTTCTGGAACCTTTCTTGAACGAACACATTTCTATGGAAAAATAGAACATATTCATATTATAGTTATAGTAGTGGGGTGTCATAATTATTTTCAGAATACCCTATGGCCCGTCAAGGGTCCTTTCATGCATTATGTTAGATATCAGGGAAAAGCGATTCTAGCTTCAAGGGGGACCCATCTTCTGATGAAAAAATGGAGATATTACCTTGTTAATTTCTGGCAATATTATTTTAGCTTTTGGTCTCAACCGTACAGGATCCATATAAACCAATTATCAAACTATTCCTTCTATTTTCTGGGGTATCTTTCAAGTGTACTAATAAATACTTCAGCAATAAAGAATCAAATGCTAGAGAACTCATATCTAATAGATACTGTTATTAATATTAACAAATTTGATACCATAGTCCCAATTATTCCTCTTATTGGATCATTGTCTAAAGCGAAATTTTGTACTGCCTCAGGGCATCCTATTAGTAAGCCGATGTGGGCTGATTTATCAGATTCTGATATTATTGATCGATTTGGTCGGATATGTAGAAATCTTTCTCATTATTATAGTGGATCTTCAAAAAAACAAAGTTTGTATCGAATAAAGTATATACTTAGACTT

>Smilax purhampuy Ruiz, maturase K (matK) gene, partial cds, voucher CIBE-011

ATCTGGTTCAAATCCTTCAATGCTGGATTCAAGATGTTCCCTCTTTACATTTATTGCGACTCTTTCTTCATAAATATCATAATTTGAATAGATTTATTACTCTGAATAAATCTATTTACGTTTTTTCAAAAGAAAATACAAGACTATTTCGCTTCCTGTATAATTCTTATGTAGCTGAATGTGAATTTTTATTTGTTTTTCTTCGTAAACAATCCTATTATTTACGATCAACATCTTCTGGAACCTTTCTTGAACGAACACATTTCTATGGAAAAATAGAACATATTCATATTATAGTTATAGTAGTGGGGTGTCATAATTATTTTCAGAATACCCTATGGCCCGTCAAGGGTCCTTTCATGCATTATGTTAGATATCAGGGAAAAGCGATTCTAGCTTCAAGGGGGACCCATCTTCTGATGAAAAAATGGAGATATTACCTTGTTAATTTCTGGCAATATTATTTTAGCTTTTGGTCTCAACCGTACAGGATCCATATAAACCAATTATCAAACTATTCCTTCTATTTTCTGGGGTATCTTTCAAGTGTACTAATAAATACTTCAGCAATAAAGAATCAAATGCTAGAGAACTCATATCTAATAGATACTGTTATTAATATTAACAAATTTGATACCATAGTCCCAATTATTCCTCTTATTGGATCATTGTCTAAAGCGAAATTTTGTACTGCCTCAGGGCATCCTATTAGTAAGCCGATGTGGGCTGATTTATCAGATTCTGATATTATTGATCGATTTGGTCGGATATGTAGAAATCTTTCTCATTATTATAGTGGATCTTCAAAAAAACAAAGTTTGTATCGAATAAAGTATATACTTAGACTT

>Smilax purhampuy Ruiz, maturase K (matK) gene, partial cds, voucher CIBE-012

TGGTTCAAATCCTTCAATGCTGGATTCAAGATGTTCCCTCGTTACATTTATTGCGACTCTTTCTTCATAAATATCATAATTTGAATAGATTTATGACTCTGAATAAATCTATTTACGTTTTTTCAAAAGAAAATACAAGACTATTTCGCTTCCTGTATAATTCTTATGTAGCTGAATGCGAATTTTTATTAGTTTTTCTTCGTAAACAATCCTATTATTTACGATCAACATCTTCTGGAACCTTTCTTGAACGAACACATTTCTATGGAAAAATAGAACATATTCATATTATAGTAGTAGGGTGTCATAATTATTTTCAGAAGACCCTATGGCCCGTCAAGGGTCCTTTCATGCATTATGTTAGATATCAGGGAAAAGCGATTCTAGCTTCAAGGGGGACCCATCTTCTGATGAAAAAATGGAGATATTACCTTGTTAATTTCTGGCAATATTATTTTAGCTTTTGGTCTCAACCGTACAGGATCCATATAAACCAATTATCAAACTATTCCTTCTATTTTCTGGGGTATCTTTCAAGTGTACTAATAAATACTTCAGCAATAAAGAATCAAATGCTAGAGAACTCATTTCTAATAGATACTGTTATTAATATTAACAAATTTGATACCATAGTCCCAATTATTCCTCTTATTGGATCATTGTCTAAAGCGAAATTTTGTACTGCCTCAGGGCATCCTATTAGTAAGCCGATGTGGGCTGATTTATCAGATTCTGATATTATTGATCGATTTGGTCGGATATGTAGAAATCTTTCTCATTATTATAGTGGATCTTCAAAAAAACAAAGTTTGTATCGAATAAAGTATATACT

>Smilax purhampuy Ruiz, RNA polymerase C (rpoC1) gene, partial cds, voucher CIBE-010

ACGAGTTGATTATTCGGGGCGTTCCGTCATTGTCGTCGGTCCTTCGCTTTCATTACATCAATGTGGATTACCTCGAGAAATAGCAATAGAGCTTTTCCAAACATTTGTAATTCGTGGTCTAATCAGACAACATCTTGCTTCTAATATAGGGATTGCTAAAAGTAAAATTCGGGAAAAAGAACCAATTGTATGGGAAATACTTCAAGAAATTATGCGGGGGCATCCTGTATTGTTGAATAGAGCGCCCACCCTGCATAGATTAGGCATACAGGCGTTCCAACCCATTTTAGTGGGGGGGTGTGCTATTTGTTTACATCCATTAGTTTGTAAGGGCTTCAATGCAGACTTTGATGGAGATCAAATGGCTGTTCATGTACCTTTATCTTTGGAAGCTCAAGCGGAGGCTCGTTTACTTATGTTTTCTCATATGAATCTCTTGTCTCCAGCTATTGGAGATCCCATTTCCGTACCAACTCA

>Smilax purhampuy Ruiz, RNA polymerase C (rpoC1) gene, partial cds, voucher CIBE-011

ACGAGTTGATTATTCGGGGCGTTCCGTCATTGTCGTCGGTCCTTCGCTTTCATTACATCAATGTGGATTACCTCGAGAAATAGCAATAGAGCTTTTCCAAACATTTGTAATTCGTGGTCTAATCAGACAACATCTTGCTTCTAATATAGGGATTGCTAAAAGTAAAATTCGGGAAAAAGAACCAATTGTATGGGAAATACTTCAAGAAATTATGCGGGGGCATCCTGTATTGTTGAATAGAGCGCCCACCCTGCATAGATTAGGCATACAGGCGTTCCAACCCATTTTAGTGGGGGGGTGTGCTATTTGTTTACATCCATTAGTTTGTAAGGGCTTCAATGCAGACTTTGATGGAGATCAAATGGCTGTTCATGTACCTTTATCTTTGGAAGCTCAAGCGGAGGCTCGTTTACTTATGTTTTCTCATATGAATCTCTTGTCTCCAGCTATTGGAGATCCCATTTCCGTACCAACTCAAGA

>Smilax purhampuy Ruiz, RNA polymerase C (rpoC1) gene, partial cds, voucher CIBE-012

ACGAGTTGATTATTCGGGGCGTTCCGTCATTGTCGTCGGTCCTTCGCTTTCATTACATCAATGTGGATTACCTCGAGAAATAGCAATAGAGCTTTTCCAAACATTTGTAATTCGTGGTCTAATCAGACAACATCTTGCTTCTAATATAGGGATTGCTAAAAGTAAAATTCGGGAAAAAGAACCAATTGTATGGGAAATACTTCAAGAAATTATGCGGGGGCATCCTGTATTGTTGAATAGAGCGCCCACCCTGCATAGATTAGGCATACAGGCGTTCCAACCCATTTTAGTGGGGGGGTGTGCTATTTGTTTACATCCATTAGTTTGTAAGGGCTTCAATGCAGACTTTGATGGAGATCAAATGGCTGTTCATGTACCTTTATCTTTGGAAGCTCAAGCGGAGGCTCGTTTACTTATGTTTTCTCATATGAATCTCTTGTCTCCAGCTATTGGAGATCCCATTTCCGTACCAACTCAAG

>Smilax purhampuy Ruiz, RNA polymerase beta subunit (rpoB) gene, partial cds, voucher CIBE-010

ACTGGGCTGGAACGCCAAACGGCTCTGGATTCGGGGCTTTCTGCTATCGCTGAACATGAGGGAAAGATCATTTATACTGATACTCACAAGATCGTTATTTCAAGTAATGGGGACACTATAAATATTCCATTAGTTATGTATCAACGTTCCAACAAAAATACTTGTATGCATCAAAAACCTCAGGTTCAACGGGGTAAATGTATTAAAAAAGGACAAATTTTAGCGGACGGTGCGGCTACAGTTGGGGGGGAACTCGCTTTAGGAAAAAACATATTAGTAGCTTATATGCCATGGGAAGGTTACAATTCTGAAGACGCAGTACTAATTAGCGAACGTCTGGTATATGAAGATATTTATACTTCTT

>Smilax purhampuy Ruiz, RNA polymerase beta subunit (rpoB) gene, partial cds, voucher CIBE-011

ACTGGGCTGGAACGCCAAACGGCTCTGGATTCGGGGCTTTCTGCTATCGCTGAACATGAGGGAAAGATCATTTATACTGATACTCACAAGATCGTTATTTCAAGTAATGGGGACACTATAAATATTCCATTAGTTATGTATCAACGTTCCAACAAAAATACTTGTATGCATCAAAAACCTCAGGTTCAACGGGGTAAATGTATTAAAAAAGGACAAATTTTAGCGGACGGTGCGGCTACAGTTGGGGGGGAACTCGCTTTAGGAAAAAACATATTAGTAGCTTATATGCCATGGGAAGGTTACAATTCTGAAGACGCAGTACTAATTAGCGAACGTCTGGTATATGAAGATATTTATACTTCTTT

>Smilax purhampuy Ruiz, RNA polymerase beta subunit (rpoB) gene, partial cds, voucher CIBE-012

ACTGGGCTGGAACGCCAAACGGCTCTGGATTCGGGGCTTTCTGCTATCGCTGAACATGAGGGAAAGATCATTTATACTGATACTCACAAGATCGTTATTTCAAGTAATGGGGACACTATAAATATTCCATTAGTTATGTATCAACGTTCCAACAAAAATACTTGTATGCATCAAAAACCTCAGGTTCAACGGGGTAAATGTATTAAAAAAGGACAAATTTTAGCGGACGGTGCGGCTACAGTTGGGGGGGAACTCGCTTTAGGAAAAAACATATTAGTAGCTTATATGCCATGGGAAGGTTACAATTCTGAAGACGCAGTACTAATTAGCGAACGTCTGGTATATGAAGATATTTATACTTCTTTT
