# Supplementary material for: Molecular barcode and morphological analysis of Smilax purhampuy Ruiz, Ecuador
Source: PeerJ. 2021 Mar 18;9:e11028. doi: 10.7717/peerj.11028 (PMC7982074; doi:10.7717/peerj.11028)
Supplement: Data S2 [file peerj-09-11028-s004.docx]

Data S2. Accession numbers of *Smilax purhampuy* Ruiz (GUAY 13117) barcode sequences at GenBank (www.ncbi.nlm.nih.gov/genbank).

| **Barcode *locus*** | **Specimen code** | **Accesion Number GenBank** |
| --- | --- | --- |
| *psbK-psbI* | CIBE-010 | MW300280 |
|  | CIBE-011 | MW300281 |
|  | CIBE-012 | MW300282 |
| *rpoB* | CIBE-010 | MT740231 |
|  | CIBE-011 | MT740232 |
|  | CIBE-012 | MT740233 |
| *rpoC1* | CIBE-010 | MT740234 |
|  | CIBE-011 | MT740235 |
|  | CIBE-012 | MT740236 |
| *atpF-atpH* | CIBE-010 | MW300277 |
|  | CIBE-011 | MW300278 |
|  | CIBE-012 | MW300279 |
| *matK* | CIBE-010 | MT740237 |
|  | CIBE-011 | MT740238 |
|  | CIBE-012 | MT740239 |
| *rbcL* | CIBE-010 | MT740240 |
|  | CIBE-011 | MT740241 |
|  | CIBE-012 | MT740242 |
| ITS2 | CIBE-010 | MT734663 |
|  | CIBE-011 | MT734664 |
